# Combined Lag Screw and Cerclage Wire Fixation for Calcaneal Tuberosity Avulsion Fractures

**DOI:** 10.1155/2018/6207024

**Published:** 2018-11-11

**Authors:** Vincenzo Giordano, Alexandre Leme Godoy-Santos, Felipe Serrão de Souza, Hilton Augusto Koch, Cesar de Cesar Netto, Stefan Rammelt

**Affiliations:** ^1^Serviço de Ortopedia e Traumatologia Prof. Nova Monteiro, Hospital Municipal Miguel Couto, Rio de Janeiro, Brazil; ^2^Hospital das Clínicas HCFMUSP, Faculdade de Medicina, Universidade de São Paulo, São Paulo, Brazil; ^3^Hospital Israelita Albert Einstein, São Paulo, Brazil; ^4^Departamento de Radiologia, Universidade Federal do Rio de Janeiro, Rio de Janeiro, Brazil; ^5^Department of Orthopedics Foot and Ankle Surgery, Hospital for Special Surgery, New York, USA; ^6^Klinik für Unfall und Wiederherstellungschirurgie, Universitätsklinikum Carl Gustav Carus, Dresden, Germany

## Abstract

Avulsion fractures of the calcaneal tuberosity represent a rare injury pattern that is caused by a powerful tension force from the Achilles tendon and is usually seen following minor trauma, especially in elderly patients. The objective of this study is to describe a surgical technique using cerclage wiring through cannulated screws in the treatment of extra- and intra-articular avulsion fractures of the calcaneal tuberosity and to present our results in a small patient's cohort. Through a 5.0 cm longitudinal skin incision over the posterolateral aspect of the calcaneus, after adequate debridement of the fracture fragments and while keeping the ankle in plantarflexion, the calcaneal tuberosity is anatomically reduced with the help of a periarticular reduction clamp and an accessory plantar longitudinal approach. Provisionally fixation is performed with K-wires. Definitive fixation is achieved with two parallel partially threaded 7.0 cannulated screws, which are positioned from the superior and posterior aspect of the tuberosity to the inferior and anterior aspect of the plantar surface of the calcaneus, and 1.5 mm cerclage wires that are pulled epiperiosteally to the plantar aspect of the calcaneus to avoid damage to local soft tissues. Alternatively, for smaller fracture fragments, two 3.5 mm partially threaded cannulated screws and 1.25 mm cerclage wires can be used. We also report the results of the procedure in a small cohort of four patients. All fractures healed in an anatomic position. There was no failure of fixation, loss of reduction, or need for secondary surgery, including hardware removal. At final follow-up, all patients had regained full plantar flexion range of motion and strength, with no gait or weight-bearing restrictions. In conclusion, the combination of cerclage wire and large diameter cannulated screws represents a promising option in the treatment of avulsion fractures of the calcaneal tuberosity, demonstrating good functional and radiographic results in our cohort of patients.

## 1. Introduction

Avulsion fractures of the calcaneal tuberosity (extra-articular or beak fractures) are rare, accounting for 1% to 3% of all calcaneal fractures [1–3]. These injuries are typically caused by a violent concentric contraction of the gastrocnemius-soleus muscle complex after a stumble or fall [1–4]. Proximal displacement of the avulsed fragment of the posterosuperior portion of the calcaneal tuberosity produces a notable weakness of the gastrocnemius-soleus complex and may lead to skin necrosis due to fragment pressure on the skin over the heel [5, 6]. The fragments are of variable size and typically include the entire insertion of the Achilles tendon [1]. They may extend into the subtalar joint and even present as open beak fractures [3, 7].

Calcaneal avulsion fractures are regularly seen in elderly patients with osteoporotic bone [1, 8]. Minor traumatic injury, delayed presentation, and irregular fragment shape should raise the suspicion of a pathologic fracture in the presence of rheumatoid arthritis or diabetic neuropathy [9, 10]. However, in younger patients, the fracture of the medial process seems to represent the first stage in the pathogenesis of a more complex injury pattern that ultimately involves the posterior tuberosity of the calcaneus [11].

Urgent anatomic reduction of these fracture patterns is necessary to restore the gastrocnemius-soleus complex, prevent further skin injury, and restore joint congruity [1, 3, 5–7, 11, 12]. Reduction is typically achieved through open exposure, and fixation is obtained with lag screws when the tuberosity fragment is sufficiently large [1, 4–6, 13]. However, many times fixation may be challenging either because the fracture fragment is small or because the patient has osteoporotic bone [10–13]. In addition, intrinsic gastrocnemius tightness may hinder reduction intraoperatively and increase the risk of screw pullout and deviation of the fragment during postoperative recovery [4, 12, 13]. Tension band wiring has been described as an alternative technique for relatively small and brittle fragments [1, 14]. Here, we describe a technique of modified tension band cerclage wiring through cannulated screws to allow for stable fixation even in osteoporotic fracture patterns.

In our experience, if there is a large posterior fragment, we prefer to fix the avulsion fractures of the calcaneal tuberosity (duck-beak and tongue fractures) with two 7.0 mm partially threaded cannulated screws and modified tension band wiring technique. Alternatively, for some cases, when the fragment is smaller, we prefer to use the same technique with two 3.5 mm partially threaded cannulated screws. The aim of this study is to present the surgical technique used in our institution for the management of extra- and intra-articular avulsion fractures of the calcaneal tuberosity.

Ethics approval was granted by our Hospital IRB/Ethics Committee (number 569/2014), and the study was registered at Clinical Trials National Register under number 0171/2014.

## 2. Surgical Technique

**Figure 1:**
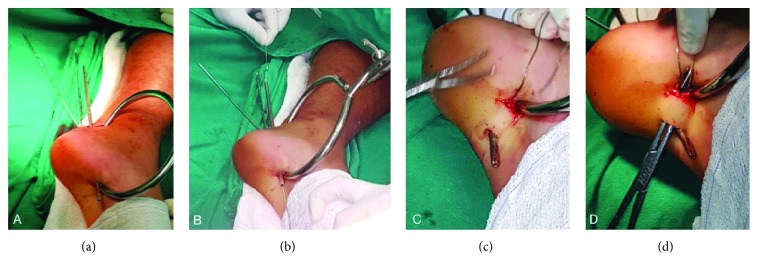
(a) With the ankle in maximal plantarflexion, a periarticular reduction clamp is applied for reduction and compression of the proximal fragment. (b) The first guide wire is overdrilled with a 4.5 mm cannulated drill bit from the upper aspect of the calcaneus tuberosity to its plantar surface, advanced through the second incision at the heel, and the cerclage wire is inserted into the cannulated drill in an antegrade fashion. (c) The wire is held in place and the drill bit is removed. (d) The cerclage wire is pulled epiperiosteally to the plantar aspect of the calcaneus to avoid damage to local soft tissues, such as plantar fascia and lateral plantar nerve.

**Figure 2:**
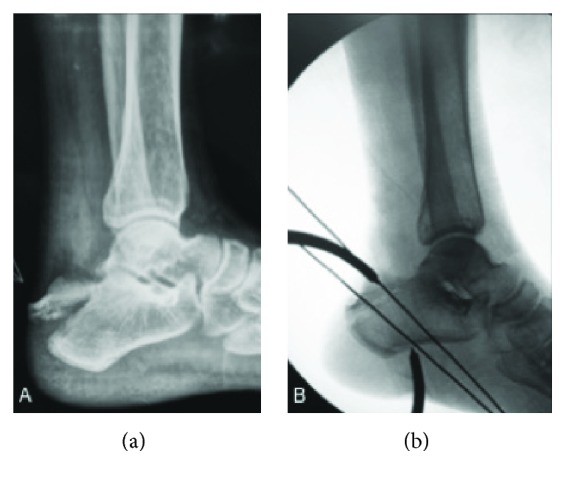
(a) Left lateral hindfoot radiograph shows an extra-articular calcaneal avulsion fracture occurring in conjunction with a fracture of the medial process (Beavis II, Squires I). Note the proximal fragment pulled cephalad by the Achilles tendon. (b) Interfragmentary compression is maintained with a periarticular reduction clamp, and provisional fixation is performed with two 2.0 mm K-wires inserted from the superior aspect of posterior tuberosity of the calcaneus.

Under general or spinal anesthesia, the patient is placed in a lateral decubitus position with the injured leg on top and the knee semiflexed. A standard radiolucent operating table is used. A pneumatic tourniquet is applied to the thigh but only inflated if deemed necessary. The fracture is approached through a 5.0 cm longitudinal skin incision over the posterolateral aspect of the calcaneus. Entrapped soft tissue, hematoma, and small intercalary fragments are removed. The displaced fracture of the calcaneal tuberosity (Figure 1(a)) is then anatomically reduced with the ankle in maximal plantarflexion. A 1.0 cm longitudinal skin incision is made on the plantar aspect of the heel, and a periarticular reduction clamp is applied for reduction and fragment compression (Figure 2). Provisional fixation is performed with two 2.0 mm K-wires that are inserted from the superior aspect of posterior tuberosity of the calcaneus, aiming to its anterior tuberosity.

Two 2.0 mm threaded guide wires for cannulated screws are inserted from the superior and posterior aspect of the tuberosity to the inferior and anterior aspect of the plantar surface of the calcaneus (Figure 1(b)). One of the previously introduced K-wires is removed to provide more space for the next steps of the procedure. The cannulated screw-measuring device is placed over the guide wires to determine appropriate screw length. One of the guide wires is then advanced through the skin incision on the plantar aspect of the heel. The first guide wire is then overdrilled with a 4.5 mm cannulated drill bit from the upper aspect of the calcaneus tuberosity to its plantar surface and advanced through the second incision at the heel. The first guide wire is removed. A 1.5 mm cerclage wire is inserted into the cannulated drill in an antegrade fashion and pulled out towards the tip of the drill bit. The wire is held in place and the drill bit is removed (Figure 1(c)).

A second 1.0 cm longitudinal skin incision is made over the plantar aspect of the heel, and the drilling procedure is repeated for the second guide wire (Figure 1(c)). The second guide wire is removed. The cerclage wire is pulled epiperiosteally to the plantar aspect of the calcaneus to avoid damage to local soft tissues, such as plantar fascia and lateral plantar nerve (Figure 1(d)).

**Figure 3:**
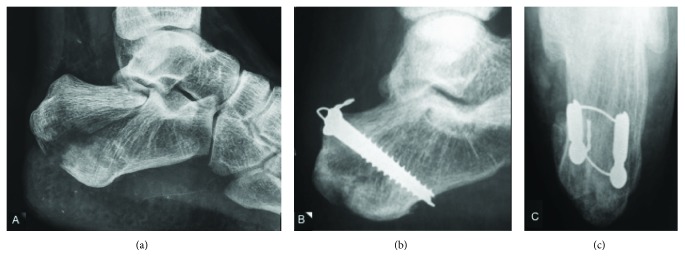
(a) Left lateral hindfoot radiograph shows an intra-articular calcaneal avulsion fracture occurring in conjunction with a fracture of the medial process (Beavis NC, Squires IV). ((b) and (c)) Lateral hindfoot and axial calcaneus radiographs show modified cerclage wire tension band technique through two 7.0 mm partially threaded cannulated screws. Observe the configuration of the wire on the axial radiograph.

The tip of the wire is then inserted into the cannulated drill bit in a retrograde fashion. The tip of the cerclage wire is advanced through the first skin incision. The cerclage wire is tensioned with a plier, and the drill bit is removed. Two 7.0 mm partially threaded cannulated screws are inserted over the cerclage wire tips. The tip of the screw should penetrate but not transgress the plantar cortex to avoid wire breakage. The wire tips are tensioned and twisted between both screw heads in a modified tension band technique (Figure 3). The skin incisions are closed with nonabsorbable interrupted sutures.

Alternatively, for smaller fragments, we prefer to use two 3.5 mm partially threaded cannulated screws with a 1.25 mm cerclage wire.

**Figure 4:**
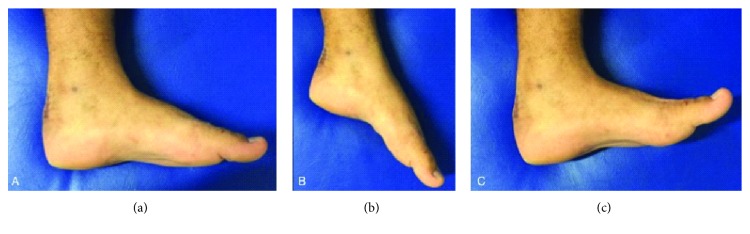
Last follow-up photographs of case 4 show a normal aspect of the hindfoot (a) with a normal plantarflexion (b) and a limitation of 10° of dorsiflexion (c). He returned fully to his prefracture activities.

Postoperatively, a Jones dressing is placed with the foot in slight ankle plantarflexion (10°-20°). Partial weightbearing is allowed as tolerated on the day after surgery with restricted dorsiflexion. Patients are generally discharged 48 hours after the surgical procedure and are scheduled for follow-up appointments until the fracture has healed and the foot function is completely regained (Figure 4).

## 3. Case Series

A retrospective review conducted between January 2015 and December 2016 identified four patients with an avulsion fracture of the posterior calcaneal tuberosity. All fractures resulted from low-energy trauma. All patients were male. None of the patients had relevant comorbidities such as diabetes, peripheral neuropathy, or rheumatoid arthritis. There were two right and two left feet. Three fractures were extra-articular and one extended into the posterior facet of the subtalar joint. We attempted to classify the fractures as proposed previously [2, 11] but neither classification proved to be applicable in all cases. Patient demographics are summarized in Table 1.

All fractures had marked superior displacement of the tuberosity fragment and considerable swelling at the superior aspect of the calcaneus. All fractures were surgically treated at a mean of two days after the injury (range, 1-3 days). There were no cases of skin necrosis, although all patients had some swelling and ecchymosis on the plantar aspect of the foot.

Clinical results were assessed with the validated Portuguese version of the American Orthopaedic Foot and Ankle Society (AOFAS) Ankle-Hindfoot score [15] at an average follow-up of 18 months. The average score was 80 (range, 76-87). All patients had regained full plantarflexion range of motion and strength. Dorsiflexion was found to be moderately restricted in all patients, with an average loss of 7° (range, 0°-10**°**).

There were no gait nor weight-bearing restrictions secondary to reduced sagittal mobility.

All fractures healed in an anatomic position. There was no failure of fixation, loss of reduction, or need for secondary surgery, including hardware removal.

## 4. Discussion

Fractures of the calcaneal tuberosity typically resulted from low-energy trauma [1–7]. A powerful concentric contraction of the gastrocnemius-soleus complex coupled with either a forced ankle dorsiflexion or a full knee extension have been implicated as the potential mechanism of injury [4]. Several authors have proposed classification systems for calcaneal tuberosity avulsion fractures, but none of them included patterns with intra-articular extension [2, 3]. Although extra-articular bony avulsions of the Achilles tendon insertion are typically present in osteoporotic patients, younger patients can present with a more complex injury pattern, where a fracture of the medial process represent the first stage of development of an injury that ultimately involves the posterior tuberosity of the calcaneus and may extend into the subtalar joint [11]. This injury pattern is different from the more frequent Essex-Lopresti tongue-type fractures that start at the crucial angle of Gissane and extend posteriorly into the posterior calcaneal tuberosity.

Surgical management of calcaneal tuberosity avulsion fractures is usually indicated for open fractures, severe skin compromise, articular step-off greater than 2.0 mm, and major extra-articular displacement (≥1.0 cm) [1–6]. Although percutaneous reduction appears ideal for these mostly extra-articular fractures, soft tissue interposition and the pull of the Achilles tendon often preclude anatomic reduction, and superior outcomes have been observed after direct visualization and open reduction of the displaced fragment [1, 4]. Numerous approaches have been proposed for stabilization of these fractures depending on the size and quality of the tuberosity fragment, including “classic” tension banding, lag screws, plates, and suture anchors with or without fracture fragment excision [1, 4–6, 10–14, 16–21].

Lag screw fixation has been advocated when the tuberosity fragment is large enough to allow the placement of at least two screws [4]. However, lag screw fixation alone may be insufficient to resist the pull forces of the Achilles tendon, particularly in the osteoporotic bone [10]. In addition, it is extremely difficult to obtain adequate lag screwing as many times both fracture orientation and distal fragment size preclude the screw threads to completely pass through the fracture line. Gitajn et al. found a 38.5% failure rate of fixation in 13 fractures fixed using cannulated lag screws alone [13]. Furthermore, a biomechanical study conducted by Khazen et al. demonstrated that lag screw fixation alone could resist approximately 250 N of tensile forces and might therefore be too weak to resist the pull of the Achilles tendon [22]. To reduce failure rate, some authors have proposed lag screw fixation followed by long-term immobilization and non-weight-bearing ambulation [4]. However, it remains unknown whether this non-weight-bearing protocol improves the clinical and functional outcomes.

The use of tension band wiring with cannulated lag screws has been recently proposed by Miyamura et al. as a better method of fixation for these fractures, avoiding displacement of the reduced fragment after fixation [20]. The authors used the technique in three elderly patients with extra-articular avulsion fractures of the calcaneal tuberosity. Headless screws were used in two patients with larger tuberosity fragments, and small fragment cannulated screws with washers were used for the patient with a smaller avulsed tuberosity fragment. They reported a mean final follow-up AOFAS score of 94. One of the potential drawbacks of this technique is the necessity for a second plantar incision, which could potentially lead to skin necrosis or painful plantar scar. However, none of these complications were reported by the authors [20].

We have been using the technique described by Miyamura et al. since 2015 with some modifications. Firstly, we expanded the indication to intra-articular fractures involving the posterior calcaneal tuberosity, such as pure tongue-type fractures and fractures of the medial process of the calcaneus with secondary avulsion fracture of the posterior tuberosity and intra-articular extension into the posterior facet of the subtalar joint, as described by Squires et al. [11]. Secondly, to reduce the risk of skin complications, we prefer to use two small longitudinal plantar incisions rather than a longer single incision. Thirdly, we use the cannulated drill bit to pass the cerclage wire, which largely facilitates the surgeon to retrogradely return the wire from the plantar surface of the calcaneus to its dorsal aspect. Finally, we use large fragment of 7.0 mm cannulated screws because they allow the passage of a large diameter cerclage wire and provide stronger fixation. For smaller fragments, we prefer the use of two 3.5 mm partially threaded cannulated screws with a 1.25 mm cerclage wire. As we have found difficulty to perform lag-screwing technique due to the size of the distal fragment, interfragmentary compression is maintained with a periarticular clamp until the cannulated screws are in place and the cerclage wiring is done. None of our patients complained of discomfort or functional limitation, and the follow-up radiographs showed that the fractures had healed in an anatomic position with no failure of fixation.

We found our modification of the technique described by Miyamura et al. to be very effective for the management of fractures of the calcaneal tuberosity, including some intra-articular patterns. It is associated with satisfactory postoperative outcomes and reduced risk of complications. The use of 7.0 mm cannulated screws and large diameter cerclage wire provides strong fixation and allows immediate partial weight-bearing and faster rehabilitation protocol.

## 5. Conclusion

Our initial results suggest that cerclage wiring through cannulated screws represents a promising option for avulsion fractures of the calcaneal tuberosity. However, due to our limited case series, there is no strong evidence to categorially recommend this technique, and we feel additional studies are required.

## Figures and Tables

**Table 1:** Patient demographics data.

Patient	Gender	Age (y)	Mechanism of injury	Beavis	Squires	Side
1	M	29	Fall down stairs	II	III	R
2	M	46	Sports injury	II	I	L
3	M	39	Fall down stairs	II	NC	R
4	M	31	Fall down stairs	NC	IV	L

Legends: M, male; y, years; NC, not classified; R, right; L, left.
